# Smith’s Bacteriology

**Published:** 1903-01

**Authors:** 


SMITH'S BACTERIOLOGY.—Lessons and Laboratory Exercises in Bacteriology. By Allen J. Smith, M. D., Professor of Pathology, University of Texas, Galveston. P. Blakiston's Sons & Co., Philadelphia, Pa. 1902. Price, $1.50, net.

This volume contains a concise and accurate "outline of technical methods, introductory to the systematic study and identification of bacteria." Its value at once appeals to the student of bacteriology, dealing as it does in a most comprehensive manner with laboratory methods and equipments—impressing most decidedly the fundamental principles, gradually leading up to detection and study of bacteria.

The subject-matter is admirably arranged—including 10 lessons and 83 exercises, well adapted to systematic study. The author has injected many original and practical methods emanating from his own fertile brain. His extensive store of knowledge and wide experience eminently fit him for the self chosen task of compiling and condensing such a valuable series of exercises. He will be many times repaid for his labor.

We predict that this laboratory manual, arranged as it is with blank pages for notes on the outcome of the various experiments for records of special instruction in technique, will occupy a prominent place in all well equipped laboratories. L.

